# Effective Preoperative Plasmapheresis Treatment of Severe Hyperthyroidism in a Patient with Giant Toxic Nodular Goiter and Methimazole-Induced Agranulocytosis

**DOI:** 10.3390/medicina56060290

**Published:** 2020-06-12

**Authors:** Katarzyna Barwinek, Danuta Gąsior-Perczak, Sławomir Trepka, Artur Szczodry, Janusz Kopczyński, Zdzisława Sitarz-Żelazna, Aldona Kowalska

**Affiliations:** 1 Collegium Medicum, Jan Kochanowski University, IX Wieków Kielc Av.19, 25-319 Kielce, Poland; barwinekkatarzyna.bk@gmail.com (K.B.); aldonako@onkol.kielce.pl (A.K.); 2 Endocrinology Clinic, Holycross Cancer Center, S. Artwińskiego St. 3, 25-734 Kielce, Poland; artur.szczodry@onkol.kielce.pl; 3 Department of Surgical Oncology, Holycross Cancer Center S. Artwińskiego St. 3, 25-734 Kielce, Poland; slavello@wp.pl; 4 Surgical Pathology, Holycross Cancer Center, S. Artwińskiego St. 3, 25-734 Kielce, Poland; janusz_kopczynski@yahoo.com; 5 Regional Blood Donation and Transfusion Center in Kielce, Jagiellońska St. 66, 25-734 Kielce, Poland; zzelazna@rckik-kielce.com.pl

**Keywords:** agranulocytosis, plasmapheresis, thyrotoxicosis, hyperthyroidism, thyroidectomy

## Abstract

Agranulocytosis is a rare but very serious complication of thyrostatic therapy. In severe hyperthyroidism, the removal of circulating thyroid hormones by plasmapheresis may be an effective therapeutic option. This report describes the therapeutic difficulties and successful preoperative treatment with plasmapheresis in a 63-year-old patient admitted to the Endocrinology Clinic with severe hyperthyroidism, during the course of giant toxic nodular goiter and agranulocytosis, which occurred after 2 weeks of taking methimazole. During hospitalization, methimazole treatment was discontinued and therapy with steroids, a beta blocker, propylthiouracil, Lugol’s solution, lithium carbonate, and antibiotics were initiated. Granulocyte colony growth stimulating factor was also used to resolve agranulocytosis. Due to the failure to achieve euthyreosis using this approach, we decided to conduct thyroid surgery, as a life-saving action, after preparation of the patient by plasmapheresis. Two plasmapheresis procedures were performed, resulting in a decrease in the concentration of free thyroid hormones. Total thyroidectomy was performed and there were no complications during surgery. We conclude that plasmapheresis may be considered as an effective alternative treatment option for the preparation of patients with hyperthyroidism for surgery, when the clinical situations prevent the use of conventional treatments for hyperthyroidism and when immediate life-saving surgery is necessary.

## 1. Introduction

Hyperthyroidism is defined as a form of thyrotoxicosis caused by excessive synthesis and secretion of thyroid hormones by the thyroid gland [1]. Treatment of hyperthyroidism depends on its cause. Three treatment methods are available: pharmacological, radioactive iodine (^131^I), and surgical. One approach is drug treatment with antithyroid drugs, which inhibit both the synthesis and the release of thyroid hormones; however, use of antithyroid drugs can be contraindicated in some patients because of the side effects, necessitating further therapies to restore euthyreosis. Agranulocytosis is the most serious side effect of antithyroid drugs, and occurs in approximately 0.2% to 0.5% of patients, usually at the beginning of treatment with methimazole or propylthiouracil; however, it can also occur later, or even during repeat therapy [1,2,3]. Agranulocytosis is an absolute indication for hospitalization and discontinuation of (and no future use of) antithyroid drugs, because of the potential risk of cross-reactivity between propylthiouracil and methimazole [1,4]. In most cases, after discontinuation of the antithyroid drug, administration of steroids and granulocyte colony growth stimulating factor (G-CSF), and appropriate treatment of concomitant infections, patients recover. Mortality in patients with agranulocytosis reaches 4% [5,6].

Patients with antithyroid drug-induced agranulocytosis are usually treated with ^131^I or surgery. Given the contraindication for the use of antithyroid drugs, the application of beta blockers, iodine preparations, lithium carbonate, and glucocorticoids are recommended for urgent preoperative preparation [7]. In cases with life-threatening thyrotoxicosis, administration of propylthiouracil can be reconsidered in patients treated with methimazole who have developed agranulocytosis, particularly if the duration of the planned treatment is short [1,8]. When other treatment options are ineffective, the final option is to prepare the patient for surgery using plasmapheresis [9]. Plasmapheresis (where ‘apheresis’ refers to removal/purification) involves extracorporeal blood filtering to remove plasma and other substances (pathogenic autoantibodies, immunocomplexes, cryoglobulins, endotoxins, lipoproteins, and primarily all thyroid hormones bound with their associated proteins), while morphotic elements are retained [10]. Plasmapheresis was first used in the treatment of hyperthyroidism in the 1970s [11] and is an effective and rapid method of thyroid hormone removal [11,12,13,14]. Here, we present the case of a patient with severe hyperthyroidism in the course of giant toxic nodular goiter and methimazole-induced agranulocytosis, which was effectively treated using plasmapheresis, allowing control over the hyperthyroidism before surgical intervention. The study was approved by the Bioethics Committee at the Świętokrzyska Chamber of Physicians on 2 June 2020 (ethic code: 8/2020-VII). We confirm that written informed consent was obtained from the participant.

## 2. Case Presentation

A 63-year-old Caucasian woman with severe thyrotoxicosis in the course of giant toxic nodular goiter was admitted to the Endocrinology Clinic of the Holycross Cancer Center in Kielce for treatment. The course of the disease was as follows: Approximately 20 years ago, the patient was diagnosed with non-toxic nodular goiter and referred for surgery. She did not agree to undergo the surgery and eventually stopped visiting the endocrinology outpatient clinic. She noticed a gradual increase in the size of the goiter, leading to symptoms of pressure manifesting as shortness of breath. Over the 4 weeks preceding her admission to hospital, she complained of nervousness, palpitations, weight loss of about 3 kg not related to changes in appetite, and difficulty in falling asleep. One week before admission to our clinic, the patient was admitted to the internal ward, due to severe shortness of breath and palpitations. She had heart failure (New York Heart Association, class II), and atrial fibrillation, with a ventricular rate of approximately 180 beats per minute, in the course of severe hyperthyroidism. New onset diabetes was also diagnosed. The following treatments were administered: methimazole (60 mg/d), beta blocker (propranolol; 120 mg/d), digitalis glycoside (digoxin; 0.25 mg/d), diuretics (furosemide; 40 mg/d), angiotensin-converting enzyme (ACE)-inhibitors (ramipril; 2.5 mg/d), anticoagulant therapy, and insulin. Due to a lack of improvement, the patient was transferred to the Endocrinology Clinic of the Holycross Cancer Center in Kielce to continue treatment and to broaden the diagnosis and determine further management options.

**Figure 1:**
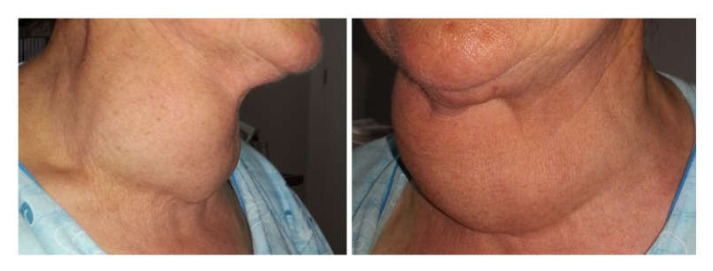
Giant nodular goiter.

On admission, the patient had a BMI of 28.4 kg/m^2^, a blood pressure of 145/90 mmHg, a body temperature of 36.5 °C, an irregular heartbeat (approximately 180 beats/min), warm skin, moist, trembling hands, and poor muscle strength. Her thyroid gland was nodular and significantly enlarged, particularly the right lobe, which descended behind the sternum, but there was no vascular murmur above the thyroid gland (Figure 1).

**Figure 2:**
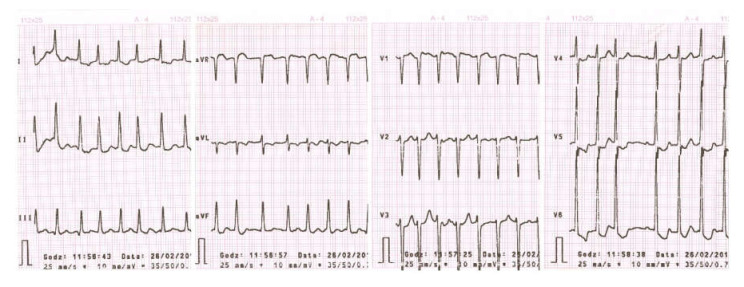
ECG (Electrocardiographic) upon presentation—visible atrial fibrillation (ventricular rate of approximately 170 beats/min). An ST segment depression of 2–3 mm detected in leads V4–V6, with 1–2 mm in leads I, II, and aVF (augmented Vector Foot).

There were no signs of lung stasis and she had minor edema of the lower limbs, around the ankles. She had no symptoms of Graves ophthalmopathy. Hormonal test findings were as follows: Thyroid stimulating hormone (TSH), 0.0009 (range, 0.3500–4.9400) µIU/mL, free thyroxine (FT_4_), 3.51 (range, 0.70–1.48) ng/dL, and free triiodothyronine (FT_3_), 4.2 (range, 1.71–3.71) pg/mL. Triiodothyronine (T3) was not assessed, and none of the following antibodies were detected: anti-thyroglobulin (anti-Tg), anti-thyroid peroxidase (anti-TPO), anti-TSH receptor (anti-TSH-TRAK), anti-thyroid-stimulating hormone receptor antibody (TRAb), and thyroid stimulating immunoglobulin (TSI). Biernacki’s reaction (erythrocyte sedimentation rate), C-reactive protein, complete blood count, and liver tests were normal. Venous plasma glucose ranged from 115 to 200 mg/dL. Electrocardiographic (ECG) examination showed atrial fibrillation, with a ventricular rate of approximately 170 beats/min. An ST segment depression of 2–3 mm was detected in leads V4–V6, with 1–2 mm in leads I, II, and augmented Vector Foot (aVF) lead (Figure 2).

**Figure 3:**
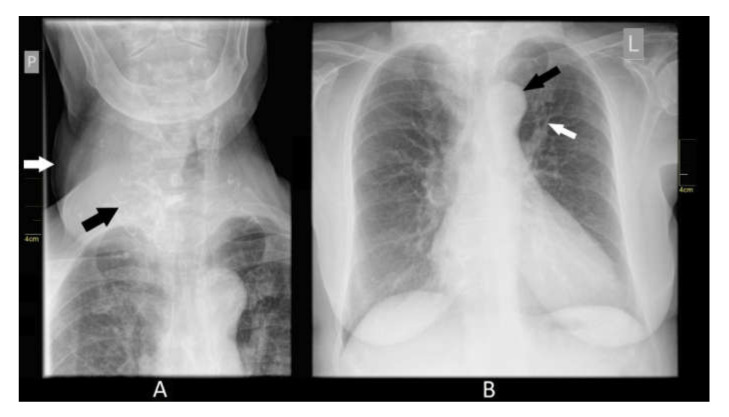
(**A**) X-ray of the neck, larynx, and nasopharynx. White arrow—enlarged right thyroid lobe; black arrow—calcifications; (**B**) Chest X-ray. White arrow—dilation of upper lobe pulmonary vessels; black arrow—thoracic aorta was elongated.

No heart chamber enlargement was detected by the transthoracic echocardiogram. There was left ventricular hypertrophy, without evident segmental contractility dysfunction. Left ventricular ejection fraction was approximately 45% (normal value, 55–70%). The Burch–Wartofsky scale score for diagnosing thyroid storm was 40, where a score of 25–44 is defined as indicating a likely impending thyroid storm [15]. The thyroid gland was heterogeneous on ultrasound, with normoechogenic lesions merging into conglomerate nodules. The largest normoechogenic lesion was 40 × 23 mm, and there were cystic nodules of up to 6 mm in the thyroid parenchyma, mainly in the right side, as well as numerous calcifications of up to 8 mm. Lobe measurement was impossible, because the lobes exceeded the measuring capability of the ultrasound probe. Local lymph nodes were not enlarged. X-ray of the neck, larynx, and nasopharynx revealed a slight shift of the trachea to the left by an enlarged thyroid lobe. There was no narrowing of the trachea. There were numerous calcifications in the soft tissues of the neck on the right side, most likely in the thyroid gland. Chest X-ray (anterolateral) showed dilation of the upper lobe pulmonary vessels. The cardiac silhouette was slightly enlarged, with a shallowed waist. The thoracic aorta was elongated (Figure 3).

During hospitalization, the doses of methimazole and β-blocker (propranolol) were increased to 80 mg/d and 180 mg/d, respectively, and administration of an oral diuretic (furosemide, 40 mg/d), digitalis glycoside (digoxin, 0.25 mg/d), ACE-inhibitors (ramipril, 2.5 mg/d), anticoagulant therapy, and insulin therapy was continued. Tachyarrhythmia slowed to approximately 120 beats/min and there were slight improvements in FT_4_ (3.17; range, 0.70–1.48 ng/dL) and FT_3_ (3.74; range, 1.71–3.71 pg/mL). During hospitalization, ultrasound-guided fine needle biopsy of the right and left thyroid nodules was performed. Benign, category II lesions were found, according to the Bethesda system [16]. On the eighth day of stay in the clinic, the patient developed a sore throat and fever of up to 38.3 °C. Laboratory tests showed leukocytes at 1.51 × 10^3^/µL (range, 4.00–10 × 10^3^/µL) and absolute neutrophil count at 0.05 × 10^3^/µL (range, 2.0–6.5 × 10^3^/µL). Agranulocytosis was diagnosed as a complication of the methimazole treatment and the methimazole treatment was discontinued. The hospital sanitary regime for people with agranulocytosis was implemented and empirical treatment with G-CSF, broad-spectrum antibiotics, and antifungal drugs were initiated. Tests conducted before the initiation of antibiotic therapy, including throat swabs, showed an increase in *Staphylococcus aureus*, Group C *Staphylococcus*, and *Candida albicans*, while blood cultures were negative. Administration of G-CSF was discontinued on day 3, when an increase in neutrophils to 6.48 × 10^3^/µL was achieved. To quickly prepare the patient for thyroidectomy, we decided to administer propylthiouracil (800 mg/d) and monitor the response in her complete blood count. Lugol’s solution (30 drops/d in four doses, once every 6 h) and lithium carbonate (1000 mg/d in four doses, once every 6 h) were administered while monitoring the serum lithium concentration. The non-selective beta-blocker (propranolol) was changed to the cardio-selective beta-1 blocker, metoprolol (Betaloc), which was administered intravenously using an infusion pump (20 mg/d), and monitored via ECG and a blood pressure monitor. Intravenous glucocorticoids (dexaven, 8 mg/d) were also used, and the oral form of digoxin was changed to an intravenous dose of 0.5 mg/d. Diuretics (furosemide, 40 mg/d), ACE inhibitor (ramipril, 2.5 mg/d), and additional treatments (anticoagulant, insulin, potassium preparation, and proton pump inhibitor) were continued. These treatments initially resulted in a poor response as shown by the following thyroid hormone levels: FT4, 2.63 (range, 0.70–1.48) ng/dL and FT3, 3.44 (range, 0.71–3.71) pg/mL. However, after 2 days, FT4 and FT3 increased to 2.73 ng/dL and 3.66 pg/mL, respectively. A complete blood count showed an absence of agranulocytosis and ECG detected atrial fibrillation (approximately 140–160 beats/min).

Given the likelihood of an impending thyroid storm, insufficient response to the pharmacological treatment, and side effects of antithyroid therapy in the form of agranulocytosis, the risk of not performing thyroid surgery was considered to be higher than the risk of performing surgery. As no other conventional treatments to restore thyroid hormone balance remained, the decision was made on day 6 to use plasmapheresis, which was performed twice; however, the second treatment was stopped due to a drop in blood pressure to 90/60 mmHg. To reduce the risk of complications, in the form of excessive bleeding during the thyroidectomy procedure, the potential deficiency of coagulation factors was compensated by the administration of fresh frozen plasma. After two plasmapheresis sessions, free thyroid hormone levels were FT4, 1.86 ng/dL (32% reduction) and FT3, 2.83 pg/mL (22% reduction). Due to the drop in blood pressure during the second procedure, no further plasmapheresis procedures were conducted, because it was concluded that her condition was the best that could be achieved and that delaying the operation threatened the recurrence of severe thyrotoxicosis. Hence, a decision was made to immediately conduct thyroid surgery, as a life-saving procedure.

**Figure 4:**
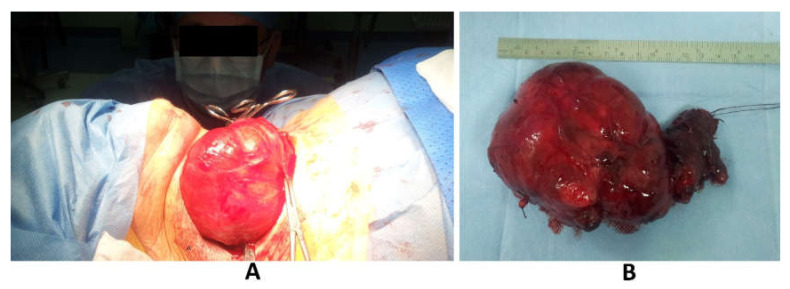
(**A**) Intra-operative findings of giant nodular goiter; (**B**) Resected thyroid specimen weighing 335 g.

The patient was referred for the surgery and underwent a total thyroidectomy within 24 h after the last plasmapheresis. There were no complications during surgery and her vital signs were stable. The duration of the procedure was 2 h. The removed thyroid gland weighed 335 g (Figure 4A,B).

**Figure 5:**
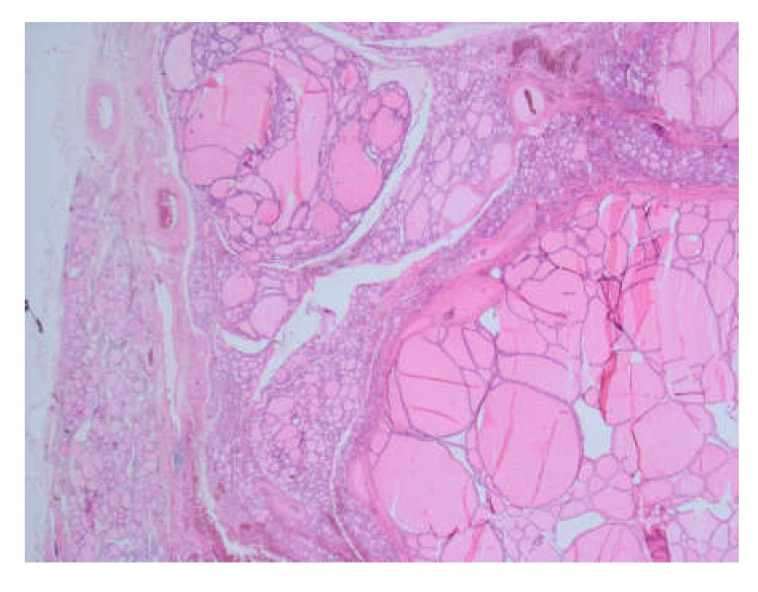
The pathology of the resected thyroid specimen showing multinodular goiter with a macrofollicular pattern, 40× magnification.

Histopathological examination confirmed benign nodular goiter (Figure 5).

After thyroidectomy, ECG showed atrial fibrillation, with a ventricular rate of approximately 140 beats/min; therefore, antiarrhythmic and circulatory therapy were continued.

The doses of steroids and insulin were gradually down-titrated. Transient hypocalcemia was observed after surgery and treated with calcium and vitamin D analogs. The patient was transferred to the Cardiology Clinic for ongoing treatment. Her sinus rhythm was restored, the cardiac treatment modified, and the insulin dose further decreased and finally discontinued during outpatient follow-up. Within two weeks of the surgery, biochemical hypothyroidism developed, requiring introduction of levothyroxine. Currently, the patient remains under endocrinological and cardiological control.

## 3. Discussion

Patients with toxic nodular goiter should be treated with ^131^I or surgery, following preparation with antithyroid drugs [17]. In patients with uncontrolled hyperthyroidism, planned surgery should be postponed until euthyreosis is obtained. In the case of an urgent procedure, the condition of the circulatory system should first be assessed, antithyroid drugs (usually methimazole) introduced, and (if there are no contraindications) a beta blocker. In addition, an inorganic or organic iodine preparation and glucocorticoids can be administered [18].

The case described here is an example of severe hyperthyroidism in the course of giant toxic nodular goiter, in which treatment with methimazole became impossible, due to poor toleration. In our patient, methimazole caused agranulocytosis and use of propylthiouracil was contraindicated because of the potential immunological cross-reaction [1,4]; however, due to the likelihood of an impending thyroid storm, we decided to use high doses of propylthiouracil (after obtaining the patient’s consent) to achieve euthyreosis. We also administered Lugol’s solution, beta blocker, lithium carbonate, and glucocorticoids to prepare the patient for thyroidectomy. This treatment gave no results, thus we decided to perform plasmapheresis.

In cases with agranulocytosis, the use of other thyreostatics is contraindicated, because the cross-reactivity between propylthiouracil and methimazole may be up to 50% [19,20]. The mechanism of the development of thyreostatic-induced agranulocytosis is not entirely clear. It is believed to be mediated by two pathogenetic mechanisms for propylthiouracil: the immune process and direct cytotoxic effects of the drug on the bone marrow [1,4,21]. However, whether these mechanisms occur with methimazole has not been fully explored. Given the lack of strong and convincing evidence that propylthiouracil-induced agranulocytosis is mediated by similar mechanisms, we decided to use propylthiouracil as a short-term treatment, in view of the impending thyroid storm, in addition to other alternative treatments for hyperthyroidism, to quickly prepare the patient for surgery. According to the American Thyroid Association (ATA), 2016 [1], this approach can be used, but it is associated with significant risk, because cross-reactivity can occur after 11 days of treatment [8,22]. There was no recurrence of agranulocytosis in our patient.

Finally, in view of the likelihood of an impending thyroid storm, we administered inorganic iodine, in the form of Lugol’s solution. Inorganic iodine effectively inhibits both the synthesis and release of thyroid hormones, which reduces their concentrations in blood much more rapidly than antithyroid drugs or glucocorticoids [23]. Another important group of drugs is beta blockers, particularly propranolol, which are recommended by the ATA because of their inhibitory effect on the conversion of T4 to T3, in addition to their inhibition of adrenergic activity [1]. Lithium carbonate reduces thyroid hormone secretion by inhibiting thyroglobulin proteolysis [24], while glucocorticoids inhibit the peripheral conversion of T4 to T3 [25]. An additional indication is the prevention of relative adreno-cortical insufficiency resulting from thyroid storm [1].

The pharmacological treatment we used did not achieve the expected results. After an initial poor response in thyroid hormone levels, FT4 and FT3 levels increased again. We did not apply the iodine contrast agents used for cholecystography (i.e., iopanoic acid and ipodate sodium), which inhibit peripheral thyroxine deiodination most strongly, as there is a lack of availability of these in our country [26]. Further, we did not use cholestyramine, an ion-exchange resin, which binds iodothyronine in the gastrointestinal tract and prevents its reabsorption, as its effects are minimal, and its efficacy has only been proven in combination with antithyroid drugs and when used for longer-duration treatment, of over 2–4 weeks [18,27,28,29].

The final option was to prepare the patient for surgery using plasmapheresis, which was introduced on day 6 after the onset of agranulocytosis, following the ineffective conventional treatment. Plasmapheresis has been described in medical literature as being able to control severe hyperthyroidism when other treatments cannot be used or are ineffective [9,11,14], as was the case in our patient. Use of a therapeutic plasmapheresis procedure in the context of treating hyperthyroidism was first described in 1970 by Ashkar et al. [11,30]. During the procedure, a significant volume of plasma is exchanged, which contains toxins and numerous proteins (including antibodies and immune complexes, among others). Thyroid hormones bound to proteins are also an important issue, as plasma exchange causes their removal from the circulation and a reduction in their intracellular concentrations [31]. In general, more than one treatment is required. It is possible to achieve a reduction in free thyroid hormones of >50% [9]. Our patient achieved reductions in free thyroid hormone concentrations of 32% and 22% for FT4 and FT3, respectively.

Plasmapheresis is an invasive procedure that carries some risk and, importantly, prior to surgery, may cause blood coagulation disorders, due to the removal of most plasma coagulation factors. After plasmapheresis, patients require a transfusion of fresh frozen plasma, to replenish the lost coagulation factors, which also provides a new pool of thyroid hormone binding proteins [14]. Therapeutic plasmapheresis, despite its apparent simplicity, also has side effects including hemolysis, anaphylactic shock or allergic reaction, infections, coagulopathy, hemorrhages, and, above all, a decrease in blood pressure [32]. Nevertheless, the effect of plasmapheresis on thyrotoxicosis is transient, lasting approximately 24–48 h, and thus surgical intervention is necessary when the patient’s condition improves [33,34]. During treatment, our patient underwent two plasmapheresis procedures: one went well, while the other had to be stopped, due to a drop in blood pressure, resulting in only moderate biochemical improvement. We decided that the patient’s condition was the best that could be achieved and that delaying surgery would threaten a recurrence of severe thyrotoxicosis; therefore, we decided to completely remove the thyroid gland.

The criteria indicating that patients with severe thyrotoxicosis should undergo thyroidectomy have not been studied in detail; hence, thyroidectomy in patients with severe thyrotoxicosis is rare. Both surgery and general anesthesia alone can cause thyroid storm [35]. In the literature, cases of undertaking thyroidectomy have been described as life-saving surgery, in cases with severe hyperthyroidism and even thyroid storm. Scolz et al. reported that the overall mortality of patients who undergo thyroidectomy as a treatment for thyroid storm is 10% [36]. In studies by Gietka-Czernel et al. and Uchida et al., thyroidectomy proved to be an effective life-saving procedure, which was necessary due to a dramatic increase in hyperthyroidism, an inability to use antithyroid drugs, and total ineffectiveness of previous treatment [37,38].

The course of the disease in our patient was very complicated, due to severe hyperthyroidism in the course of giant toxic nodular goiter, the impact of the hyperthyroidism on the heart, in the form of heart failure and rapid atrial fibrillation, and the ineffectiveness of previous treatment. Our findings suggest that total thyroidectomy should be considered in selected cases with hyperthyroidism after preparation of the patient for thyroidectomy using therapeutic plasmapheresis, if the symptoms are resistant to intensive conventional treatment. Almost 75% of patients experience a restored sinus rhythm with remission of hyperthyroidism. The chance of restoring sinus rhythm decreases with age, duration of atrial fibrillation, and the presence of other types of heart disease [39]. After a successful thyroidectomy, sinus rhythm was restored in our patient after approximately 9 days.

## 4. Conclusions

Plasmapheresis may be considered as an effective treatment option for the preparation of patients with hyperthyroidism for surgery, when the clinical situation prevents the use of conventional methods of treatment for hyperthyroidism, and when immediate life-saving surgery is necessary.
